# Comparative Analysis of Intestinal Parasitic Infections and Risk Factors Affected by the Pandemic Among Primary Schoolchildren in the Provinces of Manzini and Lubombo, Kingdom of Eswatini: A Follow-Up Study in 2019 and 2022

**DOI:** 10.1155/jotm/5950768

**Published:** 2025-08-11

**Authors:** Ai-Wen Yin, Mathobela Mbongiseni, Ting-Wu Chuang, Chia-Mei Chou, Chia-Kwung Fan

**Affiliations:** ^1^Graduate Institute of Medical Sciences, College of Medicine, Taipei Medical University, 250, Wu-Xing Street, Taipei 11031, Taiwan; ^2^Department of Molecular Parasitology and Tropical Diseases, School of Medicine, College of Medicine, Taipei Medical University, 250, Wu-Xing Street, Taipei 11031, Taiwan; ^3^Malaria & Neglected Tropical Diseases Program, Ministry of Health, P.O. Box 90, Manzini, Eswatini; ^4^Research Center of International Tropical Medicine, College of Medicine, Taipei Medical University, 250, Wu-Xing Street, Taipei 11031, Taiwan

**Keywords:** COVID-19 pandemic, Eswatini, intestinal helminthic and protozoan infections, primary schoolchildren, risk factors

## Abstract

**Background:** Intestinal parasitic infections (IPIs) are a persistent public health challenge in low- and middle-income countries, particularly among school-aged children.

**Objective:** This study aimed to compare IPI prevalence and risk factors before and after the COVID-19 pandemic in Eswatini, based on the hypothesis that pandemic-related disruptions may have influenced infection dynamics, despite no formal interventions being introduced.

**Methods:** A prospective cohort of 128 schoolchildren from Manzini and Lubombo Provinces was followed from 2019 to 2022. Stool samples were analyzed using the merthiolate–iodine–formaldehyde (MIF) method. Structured interviews assessed hygiene behaviors and household factors. Logistic regression was used to identify associations with infection risk, reported as adjusted odds ratios (aORs) with 95% confidence intervals (CIs).

**Results:** Overall, IPI prevalence remained consistent (43.0% in 2019 vs. 42.2% in 2022), with protozoan infections predominating and helminth infections remaining low (1.6% in 2019 and 2.4% in 2022, respectively). In 2022, children with only one employed parent had significantly higher odds of IPIs (aOR = 3.97; 95% CI: 1.48–10.64; *p*=0.006) and pathogenic protozoan infections (aOR = 4.33; 95% CI: 1.41–13.27; *p*=0.01). Handwashing before meals was protective in 2019 (aOR = 0.10; 95% CI: 0.02–0.58; *p*=0.01) but not significant in 2022. Notably, *Giardia intestinalis* infections declined, while *Blastocystis hominis* increased.

**Conclusion:** The stable infection rates and changing species composition suggest that pandemic-associated shifts in behavior and public health disruptions may have influenced IPIs' epidemiology. Continued surveillance and targeted hygiene interventions are needed to mitigate the burden of IPIs in schoolchildren.

## 1. Background

Intestinal parasitic infections (IPIs) remain a major public health concern, particularly in low- and middle-income countries. Globally, more than 2 billion individuals are affected, with school-aged children disproportionately burdened due to their increased exposure and vulnerability to reinfection [1]. Soil-transmitted helminths (STHs) such as *Ascaris lumbricoides*, *Trichuris trichiura*, and hookworms are prevalent in environments with inadequate sanitation and poor hygiene infrastructure [2]. Protozoan infections, including *Giardia lamblia* and *Entamoeba histolytica*, further compound the burden of IPIs, especially in regions with unsafe water sources and limited public health services [3, 4].

IPIs are associated with adverse health outcomes in children, such as anemia, malnutrition, growth retardation, and cognitive impairment, which may contribute to lower educational performance [1, 5]. In response, the World Health Organization and local health authorities have promoted strategies including preventive chemotherapy, improved sanitation, and health education to mitigate infection risks [6]. However, the sustainability of such interventions is challenged in resource-constrained settings.

The COVID-19 pandemic disrupted global health services, including programs for controlling neglected tropical diseases (NTDs). A 2021 survey reported that nearly half of participating countries experienced interruptions in NTD services, with IPI-related interventions such as mass drug administration, health education, and monitoring being severely affected [7]. The redirection of resources to pandemic response efforts and disruptions in medicine supply chains may have increased the risk of undetected or untreated parasitic infections during this period [8].

In 2019, we conducted a baseline study on IPIs among primary schoolchildren in Manzini and Lubombo Provinces of the Kingdom of Eswatini, reporting a prevalence of 40.5% [9]. The current study revisits the same cohort in 2022 to assess any changes in infection status and related risk factors influenced by the pandemic. By comparing pre- and postpandemic data, we aimed to evaluate the resilience of IPIs' control efforts and identify emerging vulnerabilities within this population.

## 2. Methods

### 2.1. Geography, Study Population, and Subject Selection in the Kingdom of Eswatini

In terms of the geography of the Kingdom of Eswatini, the country is divided into four regions based on altitude, each with its distinct landscapes. The Highveld region, which has an average altitude of 1200 m, includes the capital city of Mbabane. The Middleveld region, with an average altitude of 700 m, is home to the largest city, Manzini. The Lowveld region, with an average altitude of about 250 m, is the least populated.

**Figure 1:**
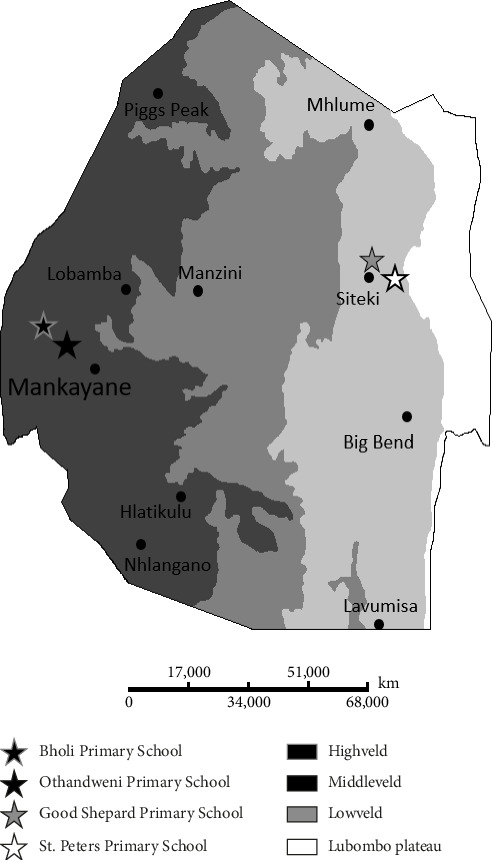
Geographical distribution of selected primary schools in the Highveld and Lowveld regions of Manzini and Lubombo Provinces, Eswatini, included in the 2019 and 2022 longitudinal study on intestinal parasitic infections (IPIs). The map illustrates the locations of four primary schools, where 128 children were followed from 2019 to 2022 to assess changes in IPI prevalence and risk factors before and after the COVID-19 pandemic. The Highveld and Lowveld regions represent distinct ecological zones that may influence transmission dynamics due to differences in altitude, climate, and infrastructure.

For the study population and subject selection, the Eswatini Malaria and NTDs Control Program selected four primary schools based on their geographical location in the Lowveld and Highveld regions (Figure 1).

### 2.2. Sample Size Justification

**Figure 2:**
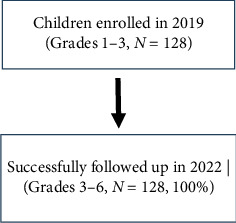
Participant flow diagram showing the 128 primary schoolchildren originally enrolled in 2019 and fully retained for follow-up in 2022. No loss to follow-up occurred.

The study cohort consists of students who originally participated in the IPIs study in 2019 when they were in Grades 1–3, and who have since progressed to Grades 3–6 in primary school in 2022. The sample size (*N* = 128) was determined by the total number of eligible students who had participated in the original 2019 baseline study and could be re-contacted and consented for follow-up in 2022 (Figure 2). As this was a longitudinal cohort design with a fixed population and no dropouts, all available participants were included to maximize power and internal consistency. While formal power calculations were not conducted, the retained sample allowed for meaningful comparison of infection trends and risk factor associations across the two time points.

### 2.3. Detection and Evaluation of Factors Contributing to the Risk of IPIs

Given our concerns about whether control of NTDs such as IPIs will be interrupted by the 2020-2021 pandemic, we initiated a comparative study. We administered the same version of the 2019 questionnaire, which included personal information, hygiene practices, and social stratification. We performed routine microscopic examinations of fecal parasites' eggs, cysts, or trophozoites using the merthiolate–iodine–formaldehyde (MIF) method (PQ ST1, Para Quick, Taiwan) [9]. For the study, each schoolchild was provided with wide-opening containers, and the collected samples were transported to the parasitology laboratory at Mbabane Government Hospital for examination. Samples that tested positive for eggs, cysts, or trophozoites using the MIF method were considered indicative of infection.

### 2.4. Statistical Analysis and Software

The demographic profiles of schoolchildren in 2019 and 2022 were compared to assess changes over the two years. Polyparasitic infections were also compared between the 2 years. Multivariate analysis was conducted using a logistic regression model. Adjusted odds ratios (aORs) and 95% confidence intervals (CIs) were estimated using logistic regression to assess associations with demographic variables, including gender, age, area of residence, parental employment status, and infection risk factors. Statistical significance was defined as a two-tailed test with a *p* value ≤ 0.05. All analyses were conducted using SAS software, Version 9.3 (SAS Institute, Cary, NC, USA). Logistic regression was performed using the “LOGISTIC” procedure.

### 2.5. Ethical Considerations

Before their children participated in epidemiological and research studies, each parent or guardian was presented with an informed consent form for approval. Approval for the study was obtained from the Eswatini Health and Human Research Review Board (EHHRRB) (SHR172/2019). Infected children received antiparasitic therapy based on the species identified during stool examination, in accordance with the national treatment guidelines of the Eswatini Ministry of Health and the WHO recommendations. Specifically, for helminth infections (e.g., *Enterobius vermicularis* and *Hymenolepis nana*), mebendazole (500 mg single dose) or albendazole (400 mg single dose) was administered. For protozoan infections such as *G. lamblia* or *E. histolytica/dispar*, children were treated with metronidazole (15 mg/kg/day in 3 divided doses for 5–7 days). All treatments were provided under the supervision of medical personnel at Mbabane Government Hospital. Children were monitored for side effects and provided follow-up care as needed.

### 2.6. Reporting Guidelines Compliance

This study adhered to the Strengthening the Reporting of Observational Studies in Epidemiology (STROBE) guidelines for cohort studies to ensure the completeness and transparency of reporting (https://www.strobe-statement.org/).

## 3. Results

A total of 128 primary schoolchildren who participated in the 2019 baseline study were successfully followed up in 2022. The overall prevalence of IPIs remained relatively stable over the three-year period, with 43.0% in 2019 and 42.2% in 2022 (Table 1). Protozoan infections accounted for the vast majority of cases in both years, while helminth infections remained consistently low (1.6%).

As shown in Table 1, protozoan infections were consistently more common than helminth infections across all demographic subgroups. Children aged ≤ 6 years exhibited the highest infection rates in both years (53.3%), with no helminth cases. Girls had higher infection rates than boys (47.2% vs. 40.0% in 2019; 49.1% vs. 37.3% in 2022). The Lowveld region saw a slight increase in infection from 41.8% to 43.3%, while the Highveld saw a marginal decline.

Parental employment status was associated with marked differences: in 2022, children with only one working parent had the highest infection rate (55.9%), compared to 28.9% for children with no working parent and 22.7% for those with both parents employed.

Children residing in brick houses showed consistent IPI rates across the 2 years (45.0% in 2019 vs. 42.7% in 2022), while a decline was observed among those living in mud houses (36.4%–25.0%). Although limited in number, children living in huts, wooden houses, or without a fixed residence had the highest infection rates, with 100% positivity in 2019 (Table 1).

As detailed in Table 2, single infections were the most prevalent overall infection type in both years (28.9% in 2019; 26.6% in 2022), while dual and multiple infections, indicative of polyparasitism, were less common. Dual infections decreased from 11.7% to 9.4%, while multiple infections increased slightly from 1.6% to 3.9%. Among pathogenic protozoa, *G. intestinalis* infections declined significantly (from 18.0% to 7.0%), while *Blastocystis hominis* infections more than doubled (from 6.3% to 14.8%). Infections with *E. histolytica/dispar* remained relatively unchanged (10.9%–10.2%). Nonpathogenic protozoa such as *E. coli* and *E. nana* showed mixed patterns of increase and decrease.

Logistic regression analysis (Table 3) revealed that in 2022, children with only one employed parent had significantly higher odds of both overall infection (aOR = 3.97; 95% CI: 1.48–10.64; *p*=0.006) and pathogenic infection (aOR = 4.33; 95% CI: 1.41–13.27; *p*=0.01), compared to children whose parents were unemployed. In 2019, washing hands before meals was significantly protective against pathogenic infections (aOR = 0.10; 95% CI: 0.02–0.58; *p*=0.01), but this association was no longer significant in 2022.

Other demographic, environmental, and behavioral variables, including gender, residence, living environment, raw food consumption, water source, pet ownership, and soil contact, did not show significant associations with infection in either year (*p* > 0.05).

## 4. Discussion

The MIF technique is a widely used parasitological method for detecting protozoan cysts, helminth eggs, and larvae in stool specimens. This method involves mixing stool samples with a fixative solution containing merthiolate (preservative), iodine (for parasite staining), and formaldehyde (for preservation), followed by concentration through centrifugation and microscopic examination of the sediment. MIF is favored for its simplicity, low cost, and long-term sample preservation, making it advantageous in field settings and particularly useful when compared to techniques such as Kato–Katz in certain epidemiological contexts [10].

This follow-up study compared the prevalence and risk factors of IPIs among the same cohort of schoolchildren in 2019 and 2022, before and after the COVID-19 pandemic, in the Kingdom of Eswatini. Despite expectations that the pandemic might significantly alter IPI transmission, the overall infection rates remained relatively stable (43.0% in 2019 vs. 42.2% in 2022), with protozoan infections continuing to dominate and helminth infections remaining low (1.6% in 2019 and 2.4% in 2022, respectively).

The consistently high prevalence of protozoan infections, particularly *G. intestinalis* and *B. hominis*, aligns with prior studies in similar contexts, where limited access to clean water and sanitation infrastructure perpetuates protozoan transmission [3]. Interestingly, *B. hominis* showed a notable increase in 2022, a pattern also observed in Lebanon following the pandemic, suggesting potential shifts in protozoan ecology or host susceptibility postpandemic [11].

Our findings also highlight dynamic associations between socioeconomic factors and infection risk. In 2022, children with only one working parent had significantly higher odds of both overall and pathogenic infections (aOR = 3.97 and 4.33, respectively), indicating that partial parental employment may relate to increased exposure, potentially due to household crowding or reduced caregiving supervision. This observation aligns with evidence suggesting that caregiver employment patterns can influence household hygiene and infection exposure [12].

Hygiene behavior played a protective role in 2019, where handwashing before meals was strongly associated with reduced IPI risk (aOR = 0.10, *p*=0.01). However, this association was not significant in 2022, possibly reflecting widespread adoption of hand hygiene during the pandemic, thereby minimizing variability in this practice [13]. While hand hygiene was maintained, environmental exposures or reinfection within households may have offset its protective effects.

The reduction in *G. intestinalis* prevalence from 18.0% in 2019 to 7.0% in 2022 is noteworthy and may reflect improvements in water source management or hygiene awareness during the pandemic. Conversely, an increase in multiple infections in 2022 suggests that although singular infections declined, children may have become more susceptible to polyparasitism, possibly due to prolonged gaps in mass deworming or surveillance services during COVID-19-related health system disruptions [7, 8].

Overall, this study underscores the resilience of protozoan transmission and highlights the importance of continuous surveillance and tailored interventions. Despite increased awareness and hygiene measures prompted by the pandemic, environmental and socioeconomic determinants remain key contributors to sustained IPI prevalence. Targeted health education and parental involvement remain critical, particularly in households with working caregivers. Reinforcing water, sanitation, and hygiene (WASH) interventions [14] alongside routine screening and treatment is essential to reduce the long-term burden of IPIs in Eswatini and similar settings.

## Figures and Tables

**Table 1:** Demographic profiling of intestinal parasitic infection among primary schoolchildren enrolled in 2019 versus 2022.

	Total	Prevalence		
		Helminth	Protozoan	All
	(*N* = 128)	*N* = 2 (%) vs. 2 (%)	*N* = 53 (%) vs. 52 (%)	*N* = 55 (%) vs. 54 (%)
Age (2019) (years)				
≦ 6	15	0 (0) vs. 0 (0)	8 (53.3) vs. 8 (53.3)	8 (53.3) vs. 8 (53.3)
7-8	75	2 (2.7) vs. 1 (1.3)	33 (44.0) vs. 30 (40.0)	35 (46.7) vs. 31 (41.3)
≧ 9	38	0 (0) vs. 1 (2.6)	12 (31.6) vs. 14 (36.8)	12 (31.6) vs. 15 (39.5)
Gender				
Boys	75	0 (0) vs. 0 (0)	30 (40.0) vs. 28 (37.3)	30 (40.0) vs. 28 (37.3)
Girls	53	2 (3.8) vs. 2 (3.8)	23 (43.4) vs. 24 (45.3)	25 (47.2) vs. 26 (49.1)
Residence				
Lowveld	67	0 (0) vs. 2 (3.0)	28 (41.8) vs. 27 (40.3)	28 (41.8) vs. 29 (43.3)
Highveld	61	2 (3.3) vs. 0 (0)	25 (41.0) vs. 25 (41.0)	27 (44.3) vs. 25 (41.0)
Employment of parents				
Both	42 vs. 22	0 (0) vs. 0 (0)	18 (42.9) vs. 5 (22.7)	18 (42.9) vs. 5 (22.7)
Either	70 vs. 68	2 (2.9) vs. 1 (1.5)	27 (38.6) vs. 37 (54.4)	29 (41.4) vs. 38 (55.9)
None	16 vs. 38	0 (0) vs. 1 (2.6)	8 (50.0) vs. 10 (26.3)	8 (50.0) vs. 11 (28.9)
Living environment				
Brick house	100 vs. 124	1 (1.0) vs. 2 (1.6)	44 (44.0) vs. 51 (41.1)	45 (45.0) vs. 53 (42.7)
Mud house	22 vs. 4	1 (4.5) vs. 0 (0)	7 (31.8) vs. 1 (25.0)	8 (36.4) vs. 1 (25.0)
Hut	1 vs. 0	0 (0) vs. 0 (0)	1 (100) vs. 0 (0)	1 (100) vs. 0 (0)
Wooden house	2 vs. 4	0 (0) vs. 0 (0)	0 (0) vs. 0 (0)	0 (0) vs. 0 (0)
Homeless	1 vs. 0	0 (0) vs. 0 (0)	1 (100) vs. 0 (0)	1 (100) vs. 0 (0)
Unknown	2 vs. 0	0 (0) vs. 0 (0)	0 (0) vs. 0 (0)	0 (0) vs. 0 (0)

**Table 2:** Distribution of polyparasitic infections in 2019 and 2022.

	Status of intestinal parasite infection						Subtotal (%)	
	Single (%)		Dual (%)		Multiple (%)			
	2019	2022	2019	2022	2019	2022	2019	2022
Area								
Lowveld								
Boys (*N* = 40)	7 (17.5)	8 (20.0)	6 (15)	3 (7.5)	0	2 (5.0)	13 (32.5)	13 (32.5)
Girls (*N* = 27)	13 (48.1)	8 (29.6)	2 (7.4)	6 (22.2)	0	1 (3.7)	15 (51.9)	15 (51.9)
Highveld								
Boys (*N* = 34)	9 (26.5)	10 (29.4)	4 (11.8)	2 (5.9)	1 (2.9)	0.0	14 (41.2)	12 (35.3)
Girls (*N* = 27)	7 (25.9)	8 (29.6)	3 (11.1)	1 (3.7)	1 (3.7)	2 (7.4)	11 (40.7)	11 (40.7)
Residence								
Lowveld (*N* = 67)	19 (28.4)	16 (23.9)	8 (11.9)	9 (13.4)	0.0	3 (4.5)	27 (40.3)	27 (40.3)
Highveld (*N* = 61)	16 (26.9)	18 (29.5)	7 (11.5)	3 (4.9)	2 (3.3)	2 (3.3)	25 (41.0)	23 (37.7)

**(*n* = 128)**	**Single (%)**		**Dual (%)**		**Multiple (%)**		**Subtotal (%)**	
	**2019**	**2022**	**2019**	**2022**	**2019**	**2022**	**2019**	**2022**

Helminth								
*E. vermicularis*	0.0	0.8 (*N* = 1)	0.0	0.8 (*N* = 1)	0.0	0.8 (*N* = 1)	0.0	2.4 (*N* = 3)
*H. nana*	1.6 (*N* = 2)	0.0	0.0	0.0	0.0	0.0	1.6 (*N* = 2)	0.0
Pathogenic protozoa								
*E. histolytica/dispar*	3.9 (*N* = 5)	1.6 (*N* = 2)	5.5 (*N* = 7)	5.5 (*N* = 7)	1.6 (*N* = 2)	3.1 (*N* = 4)	10.9 (*N* = 14)	10.2 (*N* = 13)
*G. intestinalis*	11.7 (*N* = 15)	3.9 (*N* = 5)	4.7 (*N* = 6)	1.6 (*N* = 2)	1.6 (*N* = 2)	1.6 (*N* = 2)	18.0 (*N* = 23)	7.0 (*N* = 9)
*B. hominis*	1.6 (*N* = 2)	9.4 (*N* = 12)	4.7 (*N* = 6)	4.7 (*N* = 6)	0.0	0.8 (*N* = 1)	6.3 (*N* = 8)	14.8 (*N* = 19)
Nonpathogenic protozoa								
*E. coli*	10.2 (*N* = 13)	7.8 (*N* = 10)	7.8 (*N* = 10)	6.3 (*N* = 8)	1.6 (*N* = 2)	2.3 (*N* = 3)	19.5 (*N* = 25)	16.4 (*N* = 21)
*E. nana*	0.0	0.8 (*N* = 1)	0.0	0.8 (*N* = 1)	0.0	2.3 (*N* = 3)	0.0	3.9 (*N* = 5)
*I. butschli*	0.0	1.6 (*N* = 2)	0.8 (*N* = 1)	0.8 (*N* = 1)	0.0	0.8 (*N* = 1)	0.8 (*N* = 1)	3.1 (*N* = 4)
*E. hartmanni*	0.0	0.8 (*N* = 1)	0.0	0.0	0.0	0.0	0.0	0.8 (*N* = 1)

**Table 3:** Logistic regression analysis for intestinal pathogenic parasitic infection among primary schoolchildren in 2019 versus 2022.

Variables	All (2019 vs. 2022)	Pathogenic parasites (2019 vs. 2022)
	aOR (95% CI)	aOR (95% CI)
Age (yrs)		
≦ 9	1.00	1.00
10-11	0.46 (0.12–1.8) vs. 0.82 (0.24–3.46)	0.49 (0.13–1.92) vs. 1.37 (0.34–5.49)
≧ 12	0.29 (0.07–1.26) vs. 1.25 (0.29–5.36)	0.37 (0.08–1.75) vs. 1.35 (0.29–6.23)
Gender		
Boys	1.00	1.00
Girls	0.69 (0.29–1.68) vs. 1.94 (0.79–4.42)	1.18 (0.42–3.26) vs. 2.36 (0.91–6.11)
Residence		
Lowveld	1.00	1.00
Highveld	0.86 (0.36–2.09) vs. 0.99 (0.41–2.44)	0.91 (0.33–2.55) vs. 0.66 (0.25–1.73)
Employment of parents		
None	1.00	1.00
Either	0.66 (0.17–2.59) vs. **3.97 (1.48–10.64)**	0.52 (0.1–2.64) vs. **4.33 (1.41–13.27)**
Both	0.69 (0.17–2.83) vs. 0.74 (0.19–2.91)	0.47 (0.09–2.38) vs. 1.00 (0.21–4.73)
Living environment (B)		
Others	1.00	1.00
Brick house	1.22 (0.42–3.51) vs. 0.61 (0.05–7.72)	0.41 (0.13–1.35) vs. 1.88 (0.13–27.04)
Raw meat (C)		
No	1.00	1.00
Yes	1.06 (0.37–3.04) vs. 1.35 (0.24–7.70)	1.19 (0.38–3.78) vs. 2.57 (0.43–15.18)
Raw veg (D)		
No	1.00	1.00
Yes	1.17 (0.42–3.28) vs. 1.19 (0.18–8.14)	2.41 (0.69–8.34) vs. 0.47 (0.07–3.14)
Water supply (E)		
Tap water	1.00	1.00
Well	1.17 (0.43–3.18) vs. 1.35 (0.37–5.02)	0.78 (0.24–2.52) vs. 0.58 (0.13–2.74)
Rain and others	2.13 (0.74–6.16) vs. 1.53 (0.6–3.91)	1.61 (0.48–5.39) vs. 1.47 (0.56–3.89)
Eating ground (F)		
No	1.00	1.00
Yes	0.64 (0.22–1.85) vs. 2.09 (0.91–4.81)	2.32 (0.59–9.02) vs. 1.58 (0.68–3.66)
Wash hands before a meal (G)		
No	1.00	1.00
Yes	0.25 (0.05–1.20) vs. 0.6 (0.07–5.12)	**0.1 (0.02–0.58)** vs. 1.61 (0.17–15.09)
Wash hands after toilet (H)		
No	1.00	1.00
Yes	5.72 (0.97–33.7) vs. 2.03 (0.18–22.35)	5.95 (0.75–46.9) vs. 1.15 (0.09–14.35)
Wear shoes (I)		
No	1.00	1.00
Yes	1.79 (0.69–4.64) vs. 1.25 (0.43–3.63)	3.27 (0.95–11.28) vs. 1.28 (0.41–4.03)
Touch soil (J)		
No	1.00	1.00
Yes	0.73 (0.18–3.01) vs. 1.54 (0.3–7.87)	1.29 (0.24–6.96) vs. 0.89 (0.16–5.03)
Pet (L)		
No	1.00	1.00
Yes	1.64 (0.5–5.38) vs. 0.64 (0.21–1.96)	0.54 (0.16–1.88) vs. 0.63 (0.19–2.09)

*Note:* Bold values indicate statistical significance at *p* < 0.05.

## Data Availability

The datasets generated and analyzed during the current study are not publicly available due to participant confidentiality agreements but are available from the corresponding author upon reasonable request.

## References

[1] Fauziah N., Aviani J. K., Agrianfanny Y. N., Fatimah S. N. (2022). Intestinal Parasitic Infection and Nutritional Status in Children Under Five Years Old: A Systematic Review. *Tropical Medicine and Infectious Disease*.

[2] Lebu S., Kibone W., Muoghalu C. C. (2023). Soil-Transmitted Helminths: A Critical Review of the Impact of Co-Infections and Implications for Control and Elimination. *PLoS Neglected Tropical Diseases*.

[3] Bouzid M., Kintz E., Hunter P. R. (2018). Risk Factors for *Cryptosporidium* Infection in Low- and Middle-Income Countries: A Systematic Review and meta-Analysis. *PLoS Neglected Tropical Diseases*.

[4] Fakhri Y., Daraei H., Ghaffari H. R. (2021). The Risk Factors for Intestinal *Giardia* spp Infection: Global Systematic Review and Meta-Analysis and meta-regression. *Acta Tropica*.

[5] Donkoh E. T., Berkoh D., Fosu-Gyasi S. (2023). Evidence of Reduced Academic Performance Among Schoolchildren With Helminth Infection. *International Health*.

[6] Torgerson P. R., Devleesschauwer B., Praet N. (2015). World Health Organization Estimates of the Global and Regional Disease Burden of 11 Foodborne Parasitic Diseases, 2010: a Data Synthesis. *PLoS Medicine*.

[7] Formenti B., Gregori N., Crosato V., Marchese V., Tomasoni L. R., Castelli F. (2022). The Impact of COVID-19 on Communicable and Non-Communicable Diseases in Africa: A Narrative Review. *Infezioni in Medicina, Le*.

[8] Ehrenberg J. P., Zhou X. N., Fontes G., Rocha E. M. M., Tanner M., Utzinger J. (2020). Strategies Supporting the Prevention and Control of Neglected Tropical Diseases During and Beyond the COVID-19 Pandemic. *Infectious Diseases of Poverty*.

[9] Yin A. W., Lee Y. L., Dlamini S., Maphalala G., Liao C. W., Fan C. K. (2022). Epidemiologic Investigation of Intestinal Parasite Infection and Associated Risk Factors Among Primary Schoolchildren in the Manzini and Lubombo Provinces, the Kingdom of Eswatini. *Journal of Tropical Medicine*.

[10] Gyang V. P., Chuang T. W., Liao C. W. (2019). Intestinal Parasitic Infections: Current Status and Associated Risk Factors Among School-Aged Children in an Archetypal African Urban Slum in Nigeria. *Journal of Microbiology, Immunology, and Infection*.

[11] El Achkar H., Ghandour L., Farran S., Araj G. F. (2023). Prevalence of Intestinal Parasites During Pre- and Post-COVID-19 Pandemic at a Tertiary Care Center in Lebanon. *Journal of Infection in Developing Countries*.

[12] de la Luz Galván-Ramírez M., Madriz-Elisondo A. L., Ramírez C. G. T., de Jesús Romero Rameño J., de la O Carrasco D. A., López M. A. C. (2019). Enteroparasitism and Risk Factors Associated With Clinical Manifestations in Children and Adults of Jalisco State in Western Mexico. *Osong Public Health and Research Perspectives*.

[13] Seid M., Yohanes T., Goshu Y., Jemal K., Siraj M. (2022). The Effect of Compliance to Hand Hygiene During COVID-19 on Intestinal Parasitic Infection and Intensity of Soil Transmitted Helminthes, Among Patients Attending General Hospital, Southern Ethiopia: Observational Study. *PLoS One*.

[14] Nachaiwieng W., Sanit S., Kongta N. (2024). The Impact of an Integrated Intervention Program Combining Drug Therapy with Water, Sanitation, and Hygiene (WASH) Education on Reinfection With Intestinal Parasitic Infections Among the Karen Hill Tribe in Northern Thailand. *Parasites & Vectors*.

